# Use of verbal autopsy and social autopsy in humanitarian crises

**DOI:** 10.1136/bmjgh-2017-000640

**Published:** 2018-05-03

**Authors:** Lisa-Marie Thomas, Lucia D’Ambruoso, Dina Balabanova

**Affiliations:** 1 Centre for Global Development and Institute of Applied Health Sciences, University of Aberdeen, Aberdeen, UK; 2 Umeå Centre for Global Health Research, Umeå University, Umeå, Sweden; 3 MRC/Wits Rural Public Health and Health Transitions Research Unit (Agincourt), School of Public Health, Faculty of Health Sciences, University of the Witwatersrand, Johannesburg, South Africa; 4 Department of Global Health and Development, London School of Hygiene & Tropical Medicine (LSHTM), London, UK

**Keywords:** health systems, health Policy, public health, qualitative study, review

## Abstract

**Introduction:**

Two billion people live in countries affected by conflict, violence and fragility. These are exceptional situations in which mortality shifts dramatically and in which civil registration and vital statistics systems are often weakened or cease to function. Verbal autopsy and social autopsy (VA and SA) are methods used to assign causes of death and understand the contexts in which these occur, in settings where information is otherwise unavailable. This review sought to explore the use of VA and SA in humanitarian crises, with a focus on how these approaches are used to inform policy and programme responses.

**Methods:**

A rapid scoping review was conducted on the use of VA and SA in humanitarian crises in low and middle-income countries since 1991. Drawing on a maximum variation approach, two settings of application (‘application contexts’) were selected and investigated via nine semi-structured expert interviews.

**Results:**

VA can determine causes of death in crisis-affected populations where no other registration system is in place. Combined with SA and active community involvement, these methods can deliver a holistic view of obstacles to seeking and receiving essential healthcare, yielding context-specific information to inform appropriate responses. The contexts in which VA and SA are used require adaptations to standard tools, and new mobile developments in VA raise specific ethical considerations. Furthermore, collecting and sythesising data in a timely, continuous manner, and ensuring coordination and communication between agencies, is important to realise the potential of these approaches.

**Conclusion:**

VA and SA are valuable research methods to foster evidence-informed responses for populations affected by humanitarian crises. When coordinated and communicated effectively, data generated through these methods can help to identify levels, causes and circumstances of deaths among vulnerable groups, and can enable planning and allocating resources effectively, potentially improving health system resilience to future crises.

**Key questions**

**What is already known?**

- Verbal autopsy and social autopsy (VA and SA) are research methods used to assign medical causes of death and understand the influence of social contexts on outcomes.
- They have been developed for use in settings where information systems are incomplete or absent, and where many deaths go unrecorded.
- In humanitarian crises, mortality rates shift dramatically and record-keeping and intelligence-gathering are weakened or cease to function; this can hamper data collection and obscure the true magnitude of crises.

**What are the new findings?**

- VA and SA appear to be feasible and appropriate methods for use in crisis-affected populations to gain information on levels, causes and social circumstances of death.
- Given the exceptional nature of crisis contexts, adaptations and assessments of applicability may be required. Adaptations improve operational feasibility and context specificity but limit international standardisation of methods.
- Ethical considerations related to information sharing and protection of research participants from harm, for example, by avoiding repeated interviews about deaths of close relatives, are of critical importance.
- VA and SA combined with active community involvement can inform responses that are socially appropriate and culturally acceptable.

**Key questions**

**What do the new findings imply?**

- In humanitarian crises, VA and SA can help to identify vulnerable groups, quantify levels and causes of disease, understand social and health systems contexts of ill-health, and ultimately allocate resources more effectively. Adaptations to specific contexts are likely to be necessary.
- Active involvement of communities in the process can yield data to inform locally acceptable responses, build trust between service providers and recipients, and foster an approach that centralises health equity and social justice.
- Establishing or further developing coordinated information platforms may help to improve communication and realise operational efficiencies between different agencies working in crises settings.
- Generating research evidence in collaboration with aid agencies, communities and health authorities, may help strengthen health systems and improve resilience against future crises.

## Introduction

According to the United Nations (UN), the world faces one of the largest humanitarian crises since the Second World War, with over 20 million people, mostly from Yemen, South Sudan, Somalia and Nigeria, facing famine and starvation.^1^ Approximately two billion people worldwide are thought to live in countries affected by conflict, violence and fragility.^2^

Humanitarian crises involve threats to health, widespread violence, loss of life, population displacement (internally displaced persons (IDP) or refugees), food insecurity and extensive damage to societies and economies.^3^ These are exceptional circumstances in which mortality trends can shift dramatically over short periods, and in which record-keeping and intelligence-gathering are often weakened or cease to function.^4^

The West African Ebola outbreak in 2014–2016 demonstrates the extraordinary pressures that humanitarian crises place on health systems. Two countries significantly affected were Sierra Leone and Liberia. Sierra Leone already had one of the highest maternal mortality rates in the world,^5^ and Liberia’s 14-year civil war had severe impacts on access to healthcare, as a result of the near total collapse of the health system.^6^ In both countries, Ebola combined with already high, and/or rising, levels of disease, further hampering already weakened health systems.^5^ In such situations, robust record-keeping and near-real-time intelligence provide critical information to inform the allocation of resources.^7^

Routine mortality data are typically generated from civil registration and vital statistics (CRVS) systems and provide a basis for local, regional and national planning.^8 9^ In low and middle-income countries (LMICs), CRVS development has stagnated over the past three decades.^10^ This has hampered the information basis of public services, especially for the most vulnerable, exacerbating health and social inequalities.^8^ The challenges for health surveillance and monitoring during humanitarian crises are particularly acute due to insecurity, displacement, deteriorated living conditions and impoverishment.^11^ In such situations, state information systems are often supported through coordinated aid from within and outside the country.^11^

CRVS systems are the foundation of a country’s health system.^12^ In the last decade, recognition of the importance of strengthening CRVS for health and sustainable development has gained considerable momentum.^13^ Successive key publications^14 15^ have drawn attention to the fundamental importance of regular and valid vital data, and high-profile regional fora in Africa, Asia, the Pacific and the Americas further addressed the issue.^8 16^ Subsequently, and for the first time, a decline in unrecorded deaths was observed from three-in-four deaths in 2005 to one-in-two in 2015.^17^

Verbal autopsy (VA) is an approach to determine levels and medical causes of death for people whose deaths are not registered^18^ and is a key source of vital information in lieu of incomplete CRVS. VA is currently used in over 45 LMICs,^19 20^ mostly in Health and Socio-Demographic Surveillance Systems (HDSS), Sample Vital Registration with Verbal Autopsy or as a follow-up after household surveys.^18^

VAs consist of an interview, conducted by trained fieldworkers, with final caregiver(s) of the deceased on medical signs, symptoms and circumstances immediately prior to death.^21^ Interviews are administered via paper-based questionnaires or mobile devices.^21^ Data are subsequently analysed and probable causes of deaths are assigned and coded according to the International Statistical Classification of Diseases and Related Health Problems (ICD).

Traditionally, VA data analysis and coding have been performed by physicians: ‘*Physician Certified VA’* (PCVA). However, this is expensive, time-consuming, variable within and between coders, and diverts physicians from primary roles.^20 22^ More recently, VA data entry and coding have been facilitated through automated computer algorithms ‘*Computerised Coding of VA*’ (CCVA), such as InterVA or Tariff.^23 24^ Automated interpretation has the ability to process large data sets in short periods, can relieve workload on physicians and provides a cost-effective means of implementing VA.^20^ Electronic data collection and analysis can also link with other electronic information sources improving the comprehensiveness, timeliness and accuracy of information.^20^

In the early 1990s, the WHO developed the first standard VA tools for child and maternal deaths to improve the validity and comparability of data.^25 26^ In 2007, the WHO published international VA standards for the investigation of all deaths to further harmonise data collection, comparison and analysis.^18^ In recognition of the global deficit in mortality data, the standards were updated in 2012, 2014 and 2016 into simplified, practical tools for application outside research settings.^18^ The updates also promoted the use of automated methods to improve data consistency, comparability, validity and timeliness.^18^ These developments encourage the application of VA on a wide scale to derive cause-specific mortality data without dependence on the registration of individual deaths.^27^

Social autopsy (SA) is a related method that focuses on social and health systems determinants of outcomes, providing supplementary information to that on medical cause of death.^19 28^ The method is underpinned by conceptual frameworks that consider care processes as pathways, specifically in terms of seeking, reaching and receiving care, and that identify obstacles associated with these.^28^ Standard SA tools have been developed by the Child Health Epidemiology Reference (CHERG) and the International Network for the Demographic Evaluation of Populations and Their Health (INDEPTH).^29^

While VA and SA deliver valuable information individually, when combined, a holistic view of the causes and contexts of deaths can be developed by providing accounts of deaths as biosocial phenomena. VA and SA can also assist in identifying gaps in vital statistics, enabling population disease burden estimates,^24^ as well as priority setting, benchmarking and planning,^18 30 31^ helping health systems to function more effectively.^32 33^

Humanitarian crises have profound effects on human health and health systems, and can undermine decades of development.^34^ In these situations, health systems need information about population health needs to develop effective responses.^35^ Extending the application of VA and SA into humanitarian crises, the approaches have the potential to inform resource allocation and response,^36^ and serve as an ‘interdisciplinary bridge’ connecting public health, development and humanitarian responses.^37^ The timely provision of vital information on the causes and contributing factors to outcomes via VA and SA may help to strengthen health systems and make them more resilient to shocks and crises.^38 39^

Resilient health systems are also informed by lessons learnt in the past.^33^ The capacity to prepare, respond and cope with crises comes from maintaining core functions; with information representing one of the core functions.^40^ Promoting resilience by investing in disaster recovery and through building systems (including information systems) able to absorb shocks and continue service provision is well recognised.^41 42^ Indeed the effective identification and dissemination of knowledge was recognised by the Thematic Working Group on Health Systems in Fragile and Conflict Affected States as a key mechanism to strengthen health systems capacity in crisis settings.^43^

This study aimed to explore the use of VA and SA during humanitarian crises and how the information generated informs policy and programmatic responses. ‘Responses’ here are defined as material and logistic assistance, short or long term, to people in need of help by governments or other institutions.^44^ Responses are aimed at preserving life, preventing and alleviating human suffering, and maintaining human dignity, and are based on scientific data.^44^ The specific objectives were to (1) investigate the need for cause of death information during humanitarian crises; (2) explore the application of, and adaptations to, VA and SA according to needs and circumstances in two settings, referred to as ‘application contexts’; and (3) explore the use of VA and SA to inform policy and programme responses.

## Methods

Since the use of VA and SA in humanitarian crises has not been extensively reviewed, the first step was to conduct a rapid, scoping review.^45^ We sought to develop an understanding of, for example, the importance of cause of death data and the roles of governments and humanitarian aid organisations, as well as an understanding of the use of VA and SA in different crisis settings. To highlight key features and differences of how VA and SA are applied, two application contexts were selected. Subsequently, a qualitative design was adopted, in the form of semi-structured interviews with researchers (users of or experts in VA), to elicit views on applications in real-life settings, investigating how and why the methods were used.

The review was initiated prior to the interviews, and continued concurrently with the interviewing process, so that the two elements were complementary. The literature review focused on the use of VA and SA during humanitarian crises, while the interviews sought to better understand practices of applying the methods, focusing on the two application contexts, and illustrating issues around the use of VA and SA in these contexts. Findings were analysed thematically and key lessons synthesised into research and practice recommendations.

### Literature review

A rapid, scoping review was conducted to develop an overview of VA and SA in humanitarian crises in LMICs. This approach was appropriate as it *‘aims to provide an informed conclusion on the volume and characteristics of an evidence base and a synthesis of what that evidence indicates in relation to a question’*.^46^ It allows for the identification and synthesis of the current state of understanding, for the review of available evidence in a systematic and pragmatic way.^47^ Thus, while the review employed a systematic strategy, a rapid design was selected to prioritise relatively quickly the inclusion of papers offering potential for policy relevant information. The analysis subsequently focused on key themes with relevance to VA and SA rather than conducting a full inductive analysis.^48^

**Figure 1:**
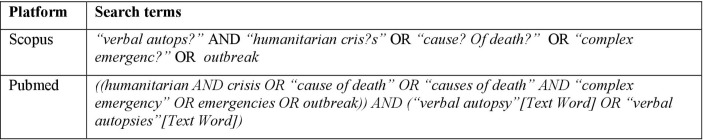
Examples of initial database search.

The following databases were searched: PubMed, Popline, Web of Knowledge, Scopus and Google Scholar. Grey literature was identified through manual searches of the portals of key agencies, for example, Centers for Disease Control and Prevention, Health Systems Global and the WHO. Keyword terms were used in combinations depending on the platform. The primary terms were verbal autopsy, social autopsy, humanitarian crisis, complex emergency, disease outbreaks, epidemic, pandemic, crisis and causes of death (figure 1). Combinations were, for example, humanitarian AND crisis OR ‘complex emergency’ AND ‘cause of death’. All searches included ‘verbal autopsy’ as text. Retrieved titles and abstracts were screened by one reviewer (LMT) for eligibility and full-text reviews were then conducted. After identifying patterns in the retrieved literature, specific terms were used, for example, specific countries with recent humanitarian crises or severe disease outbreaks.

The following inclusion criteria were applied: (1) papers accessible through internet search; (2) stated use of VA and/or SA in humanitarian crisis situations; and (3) papers written in English or German. VA and/or SA studies of chronic conditions, for example, tuberculosis and HIV, were excluded to maintain the focus on acute crises contexts. No restrictions were set on publication dates so that methodological developments could be investigated.

Data were collected from the retrieved articles using an extraction form including items on: (1) study setting; (2) information on the humanitarian crisis; (3) information on the use of VA and SA; (4) statements about difficulties and advantages of applying VA/SA; and (5) influence on informing responses. Emergent themes and subthemes were identified and compared with the findings from the interviews to identify consistently dominant themes. The lead author (LMT) conducted the analysis of both data sources, supported by coauthors (DB and LD).

### Expert interviews

#### Participant recruitment

Respondents were identified and selected according to a purposive, maximum variation approach.^49^ Selection criteria were based on having (1) professional experience in the use of VA and/or SA; (2) background of working in countries with humanitarian crises; and (3) knowledge about epidemics and/or displacement. These criteria sought to ensure that a range of viewpoints were obtained relevant to the aims and objectives. Respondents were identified through publications on VA and/or SA during crises, and through the authors’ personal and institutional networks. The snowball technique was also used,^50^ whereby respondents were asked to recommend other suitable individuals who were subsequently contacted and, if they fit the required profile, invited for interview.

Prior to the interviews, those who had agreed to participate received written information regarding the research purpose, the benefits and risks of participating and a guarantee of anonymity. Participants were informed that they were free to withdraw from the interview at any stage and for any reason. Together with the information, respondents received a consent form to be signed and returned prior to the interview.

#### Data collection and management

Drawing on preliminary findings from the literature review, a semi-structured interview guide was developed that included the following topics: (1) researchers’ experiences of applying VA and/or SA in humanitarian crises; (2) how the methods and data collected were used to inform emergency responses; and (3) respondents’ views and recommendations on the use of the methods in crisis contexts. The guide helped to elicit information on predetermined issues and allowed for comparability across the interviews. However, it was also sufficiently open to tailor the interview to respondents’ experiences in various settings, enabling them to articulate original recommendations relating to specific crisis settings. Interviews were conducted via Skype in a quiet, private location, where participants could talk freely. The interviews were audio-recorded and transcribed verbatim. During the interviews, notes were also taken. All interviews were conducted and transcribed by the lead author (LMT).

#### Analysis

Following transcription, data were organised and an inductive/deductive thematic approach to analysis was adopted. This involved using pre-existing codes derived from the literature, then revising and amalgamating these with new aspects emerging from the data.^51^ This allowed for analysis relevant to the study objectives, as well as to accommodate findings that may not have been anticipated.^52^ NVivo V.11.3.1 was used to facilitate analysis.^53^

Themes and subthemes were identified in the interview data, and the degree to which they fit with key insights from the literature, and vice versa, was assessed. Examples of themes included: use of VA and/or SA in the specific context—displacement, Ebola and others; impact of VA and/or SA evidence on responses; and adaptations of data collection tools. Checks were performed to ensure that themes and subthemes reflected the data, and thereafter themes and subthemes were mapped and interpreted to identify associations that led to descriptions and explanations of the findings.

#### Ethical considerations

Prior to the interviews, those who had agreed to participate received written information regarding the research purpose, the benefits and risks of participating and a guarantee of anonymity. Participants were informed that they were free to withdraw from the interview at any stage and for any reason. Together with the information, respondents received a consent form to be signed and returned prior to the interview. All data were anonymised and saved on a university-managed file-space.^54^

## Results

### Literature review

Thirty-one studies met the inclusion criteria. Table 1 presents the articles selected according to settings, type of crisis, year of study, and VA and/or SA tool applied. The studies took place in diverse settings: nine during conflicts,^55–63^ four during famines,^64–67^ eleven focused on displaced populations (refugees and IDP)^68–78^ and seven addressed the 2014–2016 Ebola crisis.^6 79–84^ Thirteen studies included questions or collected information solely on the social determinants of mortality.^6 55 57 59 62 66 68 73 79–83^ The earliest study identified, by Marfin *et al*, described data collection in 1991–1992.^78^ In the literature retrieved, collecting data on mortality and social determinants was described as a rapid assessment to identify vulnerable groups and evaluate where investments were needed.

**Table 1:** Key characteristics of the retrieved studies (literature review)

Author(s)	Country	Setting	Year	Tool		
				VA	SA	VA/SA
Grein *et al* 70	Angola	IDP	2001–2002	x		
Guerrier *et al* 74	Chad	IDP	2007	x		
Tomczyk *et al* 71	Chad	Refugees	2003	x		
Degomme^61^	Darfur	Conflict	2003	x		
Carrión Martín *et al* 62	DRC	Conflict	2012–2013			x
CDC^63^	DRC	Conflict	2002	x		
Coghlan *et al* 58	DRC	Conflict	2004	x		
Van Herp *et al* 57	DRC	Conflict	2001			x
Médecins Sans Frontières^59^	DRC	Conflict	2005			x
Roberts *et al* 56	DRC	Conflict	2002–2003	x		
Alberti *et al* 55	DRC	Conflict	2009			x
CDC^65^	Ethiopia	Famine	2000	x		
Salama *et al* 64	Ethiopia	Famine	2000	x		
Feikin *et al* 75	Kenya	IDP	2007–2008	x		
Kenny *et al* 79	Liberia	Ebola	2012		x	
McLean *et al* 6	Liberia	Ebola	2014			x
Morse *et al* 81	Liberia	Ebola	2014–2015		x	
Stanturf *et al* 83	Liberia	Ebola	2015		x	
Marais *et al* 82	NA	Ebola	2016		x	
Spiegel and Robinson^60^	NA	Conflict	2010	x		
CDC^69^	Nepal	Refugees	1992	x		
Marfin *et al* 78	Nepal	Refugees	1991–1992	x		
Langendorf *et al* 67	Niger	Famine	2011	x		
Hampshire *et al* 66	Nigeria	Famine	2004–2005		x	
Bartlett *et al* 68	Pakistan	Refugees	1999–2000			x
Kalter *et al* 73	Palestine	Refugees	2001–2002			x
Bower *et al* 84	Sierra Leone	Ebola	2015	x		
Polonsky *et al* 77	Somali	Refugees	2011	x		
Du Cros *et al* 76	South Sudan	Refugees	2012	x		
WHO and Federal Ministry of Health Sudan^72^	Sudan	IDP	2003	x		
Hewlett and Amolat^80^	Uganda	Ebola	2000–2001		x	
				18	6	7

CDC, Centers for Disease Control and Prevention; DRC, Democratic Republic of Congo; IDP, internally displaced persons; NA, no information available; SA, social autopsy; VA, verbal autopsy.

### Expert interviews

**Figure 2:**
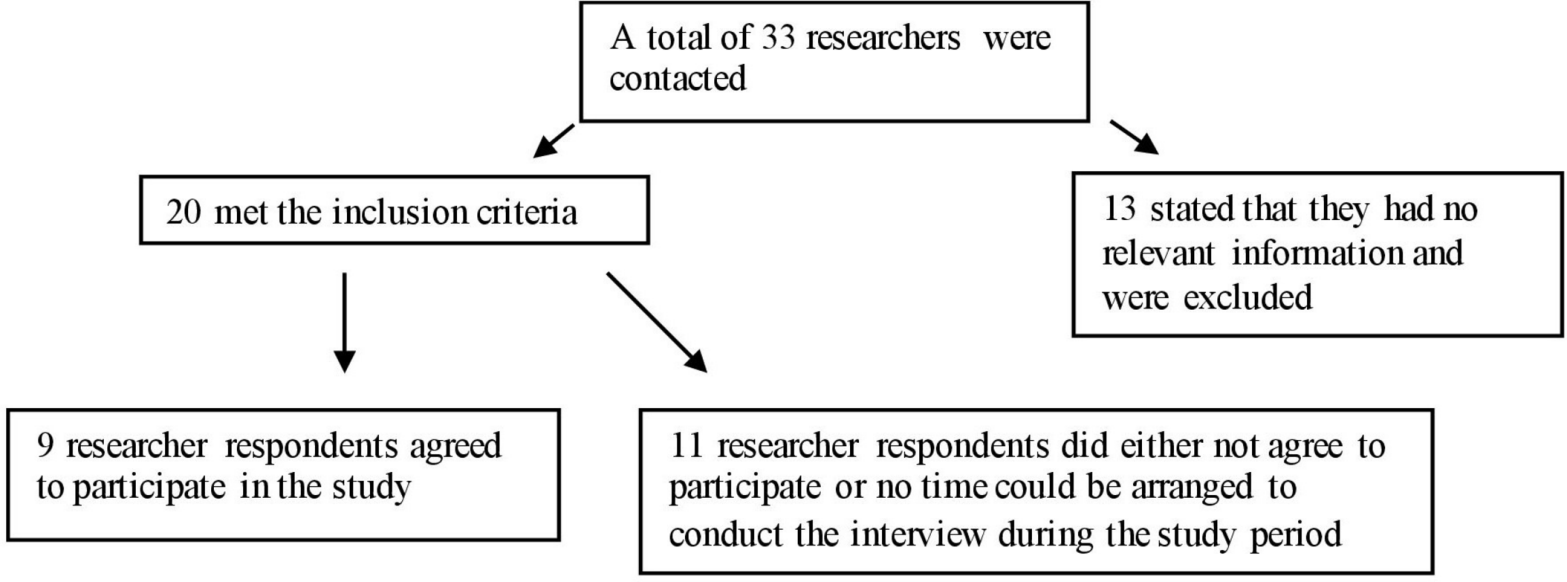
Selection of researcher respondents.

Thirty-three researchers were contacted, the majority working for universities in the UK and North America. Twenty matched the selection criteria, of which nine agreed to participate (figure 2). Among those, seven were identified through established networks and two via the snowball technique. Two respondents were also authors of papers identified in the review. The interviews took approximately 1 hour (range 30–80 min). All respondents led or were involved in major projects that took place in IDP camps, local communities and HDSS in Somalia, Uganda and Niger (table 2).

**Table 2:** Key characteristics of interview respondents (expert interviews)

Researcher	Country	Setting	Tool		
			VA	SA	VA/SA
E4	Malawi	Rural areas in Malawi	x		
E2	Niger, Cameroon, Malawi	Surveillance site			x
E9	Palestine	Towns, villages, refugee camps			x
E7	Sierra Leone	Ebola outbreak		x	
E1	Somalia	IDP camp	x		
E6	Somalia	IDP camp	x		
E8	Somalia	IDP camp	x		
E5	South Africa	HDSS site	x		
E3	Uganda	HDSS site	x		
			6	1	2

HDSS, Health and Socio-Demographic Surveillance Sites; IDP, internally displaced persons; SA, social autopsy; VA, verbal autopsy.

The following section presents the results of the literature review and interviews comparatively, according to a series of overarching themes. As the study employed a maximum variation approach, displacement and Ebola were chosen as two ‘application contexts’, that differed in terms of health systems and nature of the crisis, as well as in terms of generating and using data collected through VA and SA.

#### The need for information on mortality in crises

The recording of mortality and morbidity rates during crises was reported as necessary to estimate impacts on peoples’ lives, and to react appropriately to rapid and emerging changes in population health.^63 77 78^ Capturing age and gender differences was also reported as a means to help understand patterns compounded by the crisis (eg, incidence of direct and indirect deaths), and to identify impacts on vulnerable groups, for example, women, children or elderly people.^3 60 83^ Data collection was generally achieved through sample surveys gathering information on demographics, births and deaths, as well as on aspects such as nutritional status and vaccination coverage.^64 67 71 77^

Surveillance of mortality and morbidity was often described as hampered or neglected during crises, however.^78^ Reasons for this included resource constraints, personnel allocations to areas in need, and the destruction of health and other infrastructure.^6^ In the literature, changes in social structures and conditions, for example, displacement of people, were also described as hindering data collection.^57^ In addition, interview respondents reported that during displacements, data collection is challenged by lack of security, and that researchers have to think carefully about data requirements and purposes, and balance these against the time required to collect it.

We are focusing on child deaths, so we were able to cut out large sections of the questionnaire…. I can’t remember whether we kept those kind of more social questions or not…if we dropped them it was because of time.… It is an insecure place, so you don’t want to be in the field for a long period of time. (E1)

#### VA and SA to augment other research

Respondents described combining VA and SA with other research for indepth investigations of mortality and circumstances leading to deaths. One respondent described integrating VA into ongoing research in an IDP camp after a higher-than-expected incidence of child mortality was established and no obvious cause of death could be determined. In other cases, VA was used as a follow-up tool to investigate causes of deaths as part of ongoing studies. Elsewhere, in a study on the prevention of acute malnutrition in children, families that did not attend the distribution centre were visited and if the child had died, VA interviews were conducted.^67^

…mortality was higher than expected and they wanted to see if they could figure out why…. So, we kind of added [VA] on as an extra [to]…work that was already ongoing. (E1)

#### Application contexts: displacement

In both the literature and interviews, VA was consistently reported as feasible and appropriate for use in displacement camps. Displacement camps were described as particularly challenging environment for research due to overcrowding, high levels of in-migration/out-migration, insecurity, nutritional crises and disease outbreaks with the potential for rapid spread.^61 85^ These conditions were acknowledged to directly impede data collection and validity. Furthermore, when applied in camps, some studies reported that VA questionnaires had to be simplified and divided into traumatic/violent and non-violent deaths to identify the effects of the crisis.^58 60 61 71^

In several settings, investigations of indirect deaths (ie those caused by deteriorating social, economic and health conditions^86^) were achieved by asking questions about causes of deaths perceived as typical, or most prevailing, during the displacement.^55 58 64 69 71 72 78^ In the literature, commonly preventable causes of death during displacement were injuries, measles, (bloody) diarrhoea, (acute) respiratory infection, malnutrition and malaria.^61 69 70 72 75^ Respondents reported that conducting VA interviews in camps has to be carried out in a short timeframe after identification of the death due to the transient nature of settlements.

…the risk of households, which are all kind of temporary anyhow…either breaking down or moving around…it’s actually losing the respondents that you need in order to actually do the VA. (E1)

#### VA adaptations and mobile developments

Respondents stated that the foremost difficulty associated with conducting VA during humanitarian crises is the length and complexity of the questionnaire, and the time required to conduct interviews. This was reported to be a particular issue during the acute phase of an epidemic, when collecting data was seen as a *“luxury that time cannot afford”* (E3). Several experts described trade-offs between more rigorous approaches using standardised tools and what was possible with the time and resources available.^60^

The use of non-standard tools, with direct questions on suspected cause of death, was also reported in the literature to target infectious diseases with easily identifiable or visible clinical manifestations.^58^ Only six studies stated the use of a VA standard,^57 67 68 73 75 84^ and two described the development of modified versions.^6 71^ Other studies reported using ‘informal VA’ due to time and resources constraints,^59 70 76^ or because establishing a clinical cause of death was not a primary objective.^55 64 72 77^

A similar issue was noted by a respondent working during the 2014–2016 Ebola crisis, who stated that when conducting focus groups on causes of deaths and infection chains, efforts were made not to explicitly name Ebola. Rather, open-ended questions were asked on the types and kinds of diseases that have been endured in recent years among local residents. Other respondents held views that simply asking for perceived cause of death ought to be avoided as it was deemed inaccurate and of no real value for programme development.

…these days…very kind of approximatively, very, very…superficial questions around…what did your relative die of, are no longer, should no longer be included. (E4)

Interview respondents acknowledged that changes to international standard VA questionnaires to expedite the process have to be made carefully, in order not to reduce the validity of the information collected. Other measures reported as useful to reduce interview time were instruments that incorporate skip patterns and the use of mobile devices for data collection. Respondents generally held the view that CCVA was more operationally feasible than PCVA. It was preferred by the majority of respondents as a cheaper and faster option for use in various settings, delivering comparable results across different sites in an approach that reduces reliance on medically trained staff. However, respondents also indicated that until algorithms are further developed and easier to adapt, the involvement of local physicians in analysing VA data will continue to be necessary and important.

…both [CCVA and PCVA] are useful and the idea is to come up with algorithms because they are cheaper and can be run everywhere. (E2)

#### SA: a range of applications, labels and perceptions on utility

Not all approaches that focused on the social determinants of mortality were labelled SA, and often included a variety of methods, including structured interviews with households or focus groups with rural residents.^6 55 57 59 62 66 68 73 79–83^ Generally, the benefits of methods to gain information on local and cultural habits that contribute to deaths, for example, burial rituals and questions on community knowledge about the disease, were seen as useful in the literature and by one respondent exploring infection transmission.^6 80 81^

Furthermore, respondents reported that within the various approaches to examine the social determinants of mortality and/or disease outbreaks, there were opportunities to ask a range of broader questions on disease management, access, care-seeking behaviours and case-reporting. Respondents also recounted that items on community perceptions of health systems and on use of traditional medicine are useful.^6 59 80^ These types of questions were stated by one respondent to become especially important in the aftermath of outbreaks, to assess the impact of epidemics on health systems and care-seeking behaviours:

We were interested to know why people were trying to, in some cases, avoid having any contact with the burial teams and wanted to continue burying their own way and what they would do to protect themselves. (E7)

Views on the utility of SA were varied. In studies involving refugees and IDP, the investigation of social aspects of mortality using SA, or the active involvement of the community in research, were rarely mentioned. Where investigations on social circumstances of deaths were mentioned, they were mainly aimed at describing access to services such as water, sanitation and basic healthcare.^57 72^ Some respondents considered the collection of data on social factors to be particularly important during outbreaks. It was stated that although the main causes of death many appear obvious in these situations, indirect deaths (such as those owing to poor nutrition or non-communicable diseases (NCDs)) may become neglected. In this sense, it was stated that only through amassing knowledge on causal *and* contributory factors can policy-makers and implementers rigorously assess the situation and develop effective interventions:

Social Autopsy is so valuable because it can have that broader reach and can be tailored to […] address issues that may help people to see why it is relevant to health systems. (E3)

#### Application context: Ebola and community involvement

There were specific reports in both the literature and among interview respondents of international organisations failing to use solutions derived from local knowledge and cultural beliefs as these were perceived as risky, too localised, improvised and as having the potential to amplify spread of disease.^80^ Subsequently, however, more active engagement of communities in research was reported to provide a secure environment for people to voluntarily declare signs and symptoms of infection and seek essential medical support, in turn curbing the epidemic.^87^ More generally, the literature documenting the West African Ebola outbreaks in 2000-2001 and 2014-2016 describes similar failures in health promotion including early, active and sustained engagement of affected communities.^80 82^

More recently, community involvement has been acknowledged as essential to the design and implementation of effective responses.^87^ During the most recent Ebola outbreak, the ‘*Ebola Response Anthropology Platform*’^88^ supported a community-led approach that recognised the validity of knowledge that community members bring to the process of knowing, creating, acting on and learning from knowledge to bring about positive change.^80^ This has also been reported to help understand and identify barriers to care,^6^ as well as to detect, diagnose and treat cases.^62^ Participatory approaches help develop understandings of how social norms affect vulnerability, ill-health and the ability to access care, informing responses that are locally acceptable, feasible and effective, and that centralise health equity and social justice.^89^ One interview respondent recounted how mitigating actions undertaken by communities and communicated to international agencies over time came to be seen as substantial contributions to controlling the Ebola epidemic.

…rather brilliantly they, they used…personal protection, plastic robes, goggles and so on. And instead of dressing up the rider and the pillion passenger…they dressed the patient. (E7)

VA and SA combined with approaches that draw on participatory research principles may therefore help to connect information on mortality with local knowledge, developing evidence for action that is appropriate and acceptable.^5 90^

#### Ethical considerations of VA and SA in crises

A range of ethical considerations were identified in the literature review and interviews. Interview respondents recounted that recent mobile developments in VA incorporating automated data interpretation technologies can, in theory, provide probable causes of deaths shortly after interview, raising ethical dilemmas around disclosure of that information to VA interview respondents. The importance of careful consideration of whether and how such information is provided to relatives, and that this should be clearly expressed in an informed consent process that occurs prior to the VA interview, were noted by interview respondents.

…after getting the result and getting to know what people died of, will you go back and give them the feedback at an individual level? (E6)

While the literature consistently described the securing of informed consent prior to the VA interview,^6 62 64 67 68 70–75 77 84^ no information was reported on whether, and how, results on probable causes of death were fed back to families at an individual level. This probably reflects the facts that VA has historically been applied mainly for population-based rather than individual-level purposes, and that the administration of VA data collection on mobile devices is a relatively recent development.^31^ As mobile applications of VA become more common, however, ethical questions related to individual-level communication of probable cause of death conclusions will need to be resolved.

Further ethical aspects described in the interviews related to the interviewing of relatives who had lost multiple family members over short periods of time. Several respondents noted the additional burden placed on individuals by repeated interviews concerning the same deaths. This was reported to be, in part, due to numerous aid agencies and research groups operating in the same areas, and the difficult circumstances as mentioned above that arise in such contexts. Respondents generally held the view that field-based data collection ought to be well coordinated by one overseeing body to avoid repeatedly questioning the same people and causing distress. Respondents also noted the need for interviewer support, with training in counselling that could be provided during fieldwork, and through professional peer-to-peer fora inclusive of encouragement to talk about experiences, in order to develop coping strategies.

…they [fieldworkers] are speaking to mothers who lost more than one child, probably from the same sorts of causes…it just brings it home very raw-ly that it is the kind of context you are working in and it is just a desperate situation. (E1)

#### Informing responses

The literature review and expert interviews acknowledged the overall purpose of VA and SA methods in humanitarian contexts: that organisations and institutions need information on who, when, where and how a population is affected by a crisis to deliver essential services and to reduce the likelihood of increased morbidity, mortality and spread of disease.^57 59 60 66 72 73 83 91 92^ The literature also pointed to the use of VA and SA to contribute to advocacy and evaluation platforms for international donors, mostly giving recommendations to non-governmental organisations (NGOs) on interventions and programmes and/or for adaptations to the scope and/or focus of programmes.^57–59^

…knowing cause of death gives so much more information in terms of programme design and intervention evaluation. (E1)

Addressing population needs in this way was perceived by respondents and described in the literature to be effective only when data were available in a timely manner and on a continuous basis to track population health over time.^4 64 93^ Two respondents working in IDP camps in Somalia described how data delivered in this way helped to inform the initiation of vaccination and vitamin A supplementation programmes. This was also reflected in the literature.^78^

There were a higher number of measles deaths…we did feed that back…there is now an increased effort on measles immunisation within this area. (E1)

In another example, Van Herp *et al* 57 reported that Médecins Sans Frontières increased its operations in the war-affected rural parts of the Democratic Republic of Congo (DRC) after a survey indicated that the population exposed to violence had little or no access to healthcare. This led to a shift in emphasis from focused support in designated ‘health zones’, to direct support to the wider population.^57^

In the case of Ebola, gathering insider knowledge on illness, mortality and health-seeking behaviours among affected populations was reported to have helped design and test supportive programmes for survivors and families.^6 82^ The inclusion of items in the VA interview on adherence or non-adherence to aspects of community-based disease control protocols in cases where a patient has died was also described by the literature and respondents as a means to enhance programme design.^82^

#### The challenges of coordinated communication of information

Several respondents described how information gathered was provided to different agencies or published in scientific papers. Specific routes of rapid dissemination described included online platforms, government websites, specialised bulletins and presentations. Despite these efforts, however, insufficient and/or ineffective communication and coordination between agencies was a recurring theme in the literature.^64 71 72^ This was reportedly due to an absence of agencies with a mandate for overseeing surveillance systems in particular areas, designing programmes based on data, or coordinating diverse groups including government departments and a range of humanitarian agencies.^64^

Perhaps as a result, and despite varied efforts to facilitate and coordinate dissemination, respondents were generally unable to identify direct links between information sharing and policy/programme development, and it was perceived that impacts on decision-making may have occurred but that this could not be verified.

The interview respondents maintained, however, that dissemination on a regular, real-time basis, through platforms shared by multiple stakeholders, was of critical importance for coordinated communication of information. Both the literature and interviews also indicated the benefits of involving government officers, and specifically the Ministry of Health, in the process.^66 67 72 78^ Building partnerships with government ministries was clearly acknowledged as an important approach to promote the use of evidence for policy, planning and to build capacity for improved emergency responses.^64^

## Discussion

This study sought to explore the use of VA and SA during humanitarian crises, with a focus on its application in two crisis contexts: displaced populations and the 2014–2016 Ebola epidemic. The published literature and expert interviews provided information on how VA and SA tools are adapted to the special needs and circumstances in specific application contexts, and on the operational and organisational challenges faced. Finally, it sought to explore how information is collected and synthesised to inform policy and programmatic responses.

The analysis revealed that VA and SA methods are used both as stand-alone approaches, and in conjunction with surveys or other data collection methods. Among respondents, the methods are perceived as valuable tools that can be used to ascertain cause of death and the circumstances that have contributed to it. However, the extraordinary circumstances and challenges during humanitarian crises, for example, changing living conditions, impose particular challenges, often impairing data collection and validity.

Many VA and SA practitioners described adaptations to standard tools given the exceptional circumstances and contexts of their application.^60^ However, there was agreement that these modifications should not be made on an *ad hoc* basis but rather developed to ensure cross-country comparability with regard to the WHO guidance on VA.^18^ This was perceived as particularly important when the methods are used routinely to support operational work as vulnerable populations need special protection from exploitation caused by unnecessary exposure to humans during the research processes.^94^

Furthermore, the benefits of going beyond how many people have died and capturing the ‘why’ and the ‘how’ appears to be increasingly underpinning a shift in mortality surveys.^6 61 79 81^ Asking questions solely about infectious diseases or probable cause of death is generally not recommended as it only measures the ‘*tip of the iceberg*’ and omits valuable information,^61^ potentially misdirecting policy responses.

The literature and interviews revealed that the distribution of information on causes of death during disease outbreaks can be particularly problematic. For example, the collection of cause of death data with VA during the Ebola outbreak was hampered due to the perception that Ebola was the most prevalent cause of death in the affected population. It received the most attention in terms of resources, thereby neglecting other conditions, for example, NCDs. It is therefore important that information systems are fit-for-purpose, informing strategies and interventions to prevent and treat communicable as well as non-communicable diseases, as both have adverse consequences for the health and well-being of crisis-affected populations.^95^ This view is substantiated in calls for sustained efforts to address the growing, double burden of emergencies and NCDs, including among people affected by humanitarian crises.^95 96^

In crises situations, the benefits and demands of research have to be carefully considered.^97^ Acknowledging the operational and organisational challenges, timely and continuous data collection using standardised and internationally verified tools, and the involvement of the community in the process were reported to confer important benefits. This can inform the cultural appropriateness of responses by NGOs, governments and other organisations seeking to implement and adapt interventions and programmes to improve and safeguard population health.^77 98^ Similarly, building relationships with government departments in the research process may be beneficial to inform policy and planning and make sustainable changes to the use of research intelligence in the future.^64^

VA data analysed with automated methods are reported as one of the most promising developments for the generation of timely and comprehensive information.^99^ While the use of PCVA was not disregarded completely, automated data interpretation methods are seen as a valuable, consistent and cost-effective approach.^18^ It is acknowledged that automated methods require further consideration, development and training for researchers, to be able to generate accurate, context-specific information to inform the development of effective programmes.^100^ Additionally, the ethical dilemmas of whether and how to feed back probable cause of death conclusion(s) to interview respondents(s) at the time of VA interview urgently require further consideration and resolution.

SA methods are increasingly used. These are more varied and inclusive of combinations of SA and qualitative methods, which are not always labelled SA. In one example stated by a respondent, SA was conducted as part of focus group discussions to trace infection chains and understand community behaviours during the Ebola outbreak. During displacements, SA is less common, however. Insecurity, unstable living conditions and a need for rapid information gathering can impede the longer time needed for data collection, limiting the use of SA methods in these situations.

Nevertheless, our study suggested that the assessment of cultural values and norms influencing health, as well as other factors that may constrain access to, and availability of, care in camps is valuable and should not be neglected, especially post-emergency.^68 76^ In this sense, SA is a valuable approach and when conducted in conjunction with VA is perceived as able to deliver a more holistic view on the effectiveness of interventions and their uptake.^28 101^ For the wide-scale adoption and use of SA, however, further development of the methodology and some degree of standardisation of the instrument are recommended in the literature.^28 29^

Our study also suggests a range of benefits to community involvement in VA and SA in crisis settings, and there may be merit in it becoming a standard element of VA and SA application. Data collected with and for communities that captures comprehensively the realities of people’s daily lives can provide veracious and granular information to address barriers to access to healthcare. Community involvement can also inform remedial action that tackles underlying deficiencies in health systems, as well as the social determinants of health and health inequalities.^89^ Involving the community through participatory approaches has the potential to enhance the generation of shared knowledge, identify obstacles that may obstruct the uptake of essential interventions at community level, and build trust between providers and recipients of services.

The evidence suggests that data collected with VA and SA during and after a crisis can present opportunities for initiating health system reforms and rapid local interventions.^102^ Instances were identified where data gathered had a substantial impact, for example, a measles immunisation programme brought about by robust evidence on the magnitude of mortality rates.^78^ Acknowledging the considerable operational, organisational and situational challenges, data gathering that is coordinated, communicated and well managed by the organisations involved can improve impact on decision-making, programme implementation and evaluation.

The collection of data and implementation of interventions among agencies leading emergency responses in humanitarian crises has been characterised by absent and/or outdated leadership and that is often centralised and bureaucratic.^87^ According to the UN, this can lead to ineffective communication, coordination and a loss or inaccurate interpretation of valuable information, thereby limiting effective planning, delivery and evaluation of programmes.^98^

The study found limited evidence on the mechanisms through which information is disseminated. The majority of studies focused on making recommendations for implementing or scaling up discrete interventions. Further developments in platforms and systems that coordinate data collection, the upload of information and information management are therefore critical elements to respond effectively to emerging health threats and to trigger timely responses.^98^ Such platforms could include pre-crisis information on the local population, disease burden and on crisis-related risk factors, as well as health service functionality. Information that is available in working and local languages, accessible to all actors, and based on close collaboration and partnerships with stakeholders including government departments, officials and affected communities is further recommended.^76 98^

Effective and coordinated dissemination is clearly beneficial to build better response capacities, making interventions more efficient, effective and sustainable.^87 98^ Many of these points represent an arguably ideal case and may not be immediately feasible given the constraints on resources and time. It is also acknowledged that shifts in organisational cultures, particularly among aid agencies, and with regard to addressing bureaucracy and democratising information processes, are also required. Given the increasing investment by governments and donors in data collection and aggregation,^59 72 79^ there is scope for progressive realisation of these principles over time as health systems recover and adapt functions and capacities following crises.

Based on these findings, a series of recommendations are presented in table 3.

**Table 3:** Recommendations for use of VA and SA in humanitarian crises

Data	Data on morbidity, mortality and social determinants collected on a timely and continuous basis during crises, in partnership with key actors and communities to effectively inform policy and programmatic responses.
VA	Automated VA methods further developed and adapted in accordance with the crisis setting where they are used, and with reference to guidance on international standards, to obtain valid, comparable and context-specific information.
SA	Methodology and questionnaires further developed, with standardisation where possible to develop or adjust interventions and help decision-making and resource allocation cognisant of social and health systems contexts of outcomes.
VA and/or SA in combination with other data collection methods	Integrating VA and/or SA with other methods for data collection can provide a more holistic understanding of the situation while safeguarding scarce resources.
Community involvement	Community involvement in the research process to develop responses tailored to specific cultural and social norms, lived realities and social injustices, enhancing appropriateness, acceptability, and ultimately effectiveness and efficiency of responses.
Ethical considerations	Ethical considerations of protecting research participants from harmful consequences (eg, distress from repeated interviews on deaths of relatives) and around full and informed consent to participate in the research process require particular attention.
Coordinated communication	Oversight of activities by a single body is important for improving coordination and synergies across agencies. Information sharing platforms to enhance communication and coordination in crisis settings and make responses more efficient.

SA, social autopsy; VA, verbal autopsy.

## Conclusion

This study examined how VA and SA are applied to provide information on the levels, causes and circumstances of mortality during humanitarian crises to inform policy and programmatic work by humanitarian agencies and governments. The literature and expert opinion suggest that standardised data collection and the involvement of the community can deliver verified, comparable information, as well as identify vulnerable groups and local barriers to access and quality in healthcare. There was a distinct trade-off between adapting VA and SA to specific contexts and preserving standardisation for comparability purposes. Integrating VA and SA with other methods can also be advantageous to build enhanced understandings of the situation while safeguarding research resources. Given that circumstances leading to death among population subgroups in crisis situations are considerably variable and dynamic, VA and SA need to be carefully targeted, and decisions made about what information is required and how it can be obtained. Ethical considerations related to the protection of individuals, from further harm and distress, are especially pertinent. Finally, data collection, analysis and dissemination that are coordinated and communicated through appropriate and authorised platforms may have the potential to yield benefits by informing aid agencies, governments and communities alike. This is recommended to promote effective, shared responses and build resilient health systems in the longer term.
