# Correction: *Use of verbal autopsy and social autopsy in humanitarian crises*

**DOI:** 10.1136/bmjgh-2017-000640corr1

**Published:** 2018-06-09

Thomas L-M, D’Ambruoso L, Balabanova D. Use of verbal autopsy and social autopsy in humanitarian crises. *BMJ Glob Health* 2018;3:e000640. doi: 10.1136/bmjgh-2017-000640.

This article has been corrected since it first published. The author affiliations have been corrected as follows:

Lisa-Marie Thomas (1), Lucia D’Ambruoso (1,2,3), Dina Balabanova (4)

1. Centre for Global Development and Institute of Applied Health Sciences, University of Aberdeen, Aberdeen, UK.
2. MRC/Wits Rural Public Health and Health Transitions Research Unit (Agincourt), School of Public Health, Faculty of Health Sciences, University of the Witwatersrand, Johannesburg, South Africa.
3. Umeå Centre for Global Health Research, Umeå University, Umeå, Sweden.
4. Department of Global Health and Development, London School of Hygiene & Tropical Medicine (LSHTM), London, UK.

